# A decade of change towards Value-Based Health Care at a Dutch University Hospital: a complexity-informed process study

**DOI:** 10.1186/s12961-024-01181-z

**Published:** 2024-08-05

**Authors:** Veerle van Engen, Martina Buljac-Samardzic, Rob Baatenburg de Jong, Jeffrey Braithwaite, Kees Ahaus, Monique Den Hollander-Ardon, Ingrid Peters, Igna Bonfrer

**Affiliations:** 1 Erasmus School of Health Policy & Management, Erasmus University Rotterdam, Rotterdam, The Netherlands; 2 Department of Otorhinolaryngology and Head and Neck Surgery, Erasmus Medical Centre, Rotterdam, The Netherlands; 3 Centre for Healthcare Resilience and Implementation Science, Australian Institute of Health Innovation, Macquarie University, Sydney, Australia; 4 Department of Quality and Patient Care, Erasmus Medical Centre, Rotterdam, The Netherlands

**Keywords:** Value-Based Health Care, Patient reported outcome measures, Change, Implementation, Strategy, Complexity, Institutionalization, Process, Hospital, Qualitative

## Abstract

**Background:**

While healthcare organizations in several countries are embracing Value-Based Health Care (VBHC), there are limited insights into how to achieve this paradigm shift. This study examines the decade-long (2012–2023) change towards VBHC in a pioneering Dutch university hospital.

**Method:**

Through retrospective, complexity-informed process research, we study how a Dutch university hospital’s strategy to implement VBHC evolved, how implementation outcomes unfolded, and the underlying logic behind these developments. Data include the hospital’s internal documents (*n* = 10,536), implementation outcome indicators (*n* = 4), a survey among clinicians (*n* = 47), and interviews with individuals contributing to VBHC at the hospital level (*n* = 20).

**Results:**

The change towards VBHC is characterized by three sequential strategies. Initially, the focus was on deep change through local, tailored implementation of multiple VBHC elements. The strategy then transitioned to a hospital-wide program aimed at evolutionary change on a large scale, emphasizing the integration of VBHC into mainstream IT and policies. Recognizing the advantages and limitations of both strategies, the hospital currently adopts a “hybrid” strategy. This strategy delicately combines deep and broad change efforts. The strategy evolved based on accumulated insights, contextual developments and shifts in decision-makers. The complexity of change was downplayed in plans and stakeholder communication. By the end of 2023, 68 (sub)departments engaged in VBHC, enabled to discuss patients’ responses to Patient Reported Outcomes Measures (PROMs) during outpatient care. However, clinicians’ use of PROMs data showed limitations. While pioneers delved deeper into VBHC, laggards have yet to initiate it.

**Conclusions:**

VBHC does not lend itself to linear planning and is not easily scalable. While there appears to be no golden standard for implementation, blending local and larger-scale actions appears advantageous. Local, deep yet harmonized and system-integrated changes culminate in large scale transformation. Embracing complexity and focusing on the ultimate aims of (re)institutionalization and (re)professionalization are crucial.

**Supplementary Information:**

The online version contains supplementary material available at 10.1186/s12961-024-01181-z.

## Background

Many international health systems are moving towards Value-Based Health Care (VBHC) [1], a concept introduced by Porter and Teisberg in 2006. VBHC aims to transform traditional volume-centric care systems into value-driven models, where “value” is defined as the ratio between outcomes that matter to a patient and the costs required to attain these outcomes throughout the entire care cycle [1, 2]. Despite widespread interest in VBHC [3, 4], insights into its implementation in hospital settings remain scarce [3]. This gap complicates efforts and potentially compromises outcomes as hospitals may need to develop their change strategies from scratch.

VBHC’s healthcare reform involves the implementation of six elements outlined in the “value agenda” (see Box 1) [5]. Based on Dutch experiences, this agenda has been expanded, amongst others to include a focus on value-based quality improvement (addition 1) and on discussing value with patients (addition 2) (see Box 1) [6]. To support these activities and measure outcomes (element 2), Patient-Reported Outcome Measures (PROMs) have gained significant attention. PROMs contain structured questions that enable patients to self-assess and report on their symptoms, functioning, and well-being, often measured through surveys [7, 8], requiring enabling IT (element 6).

**Box 1:**
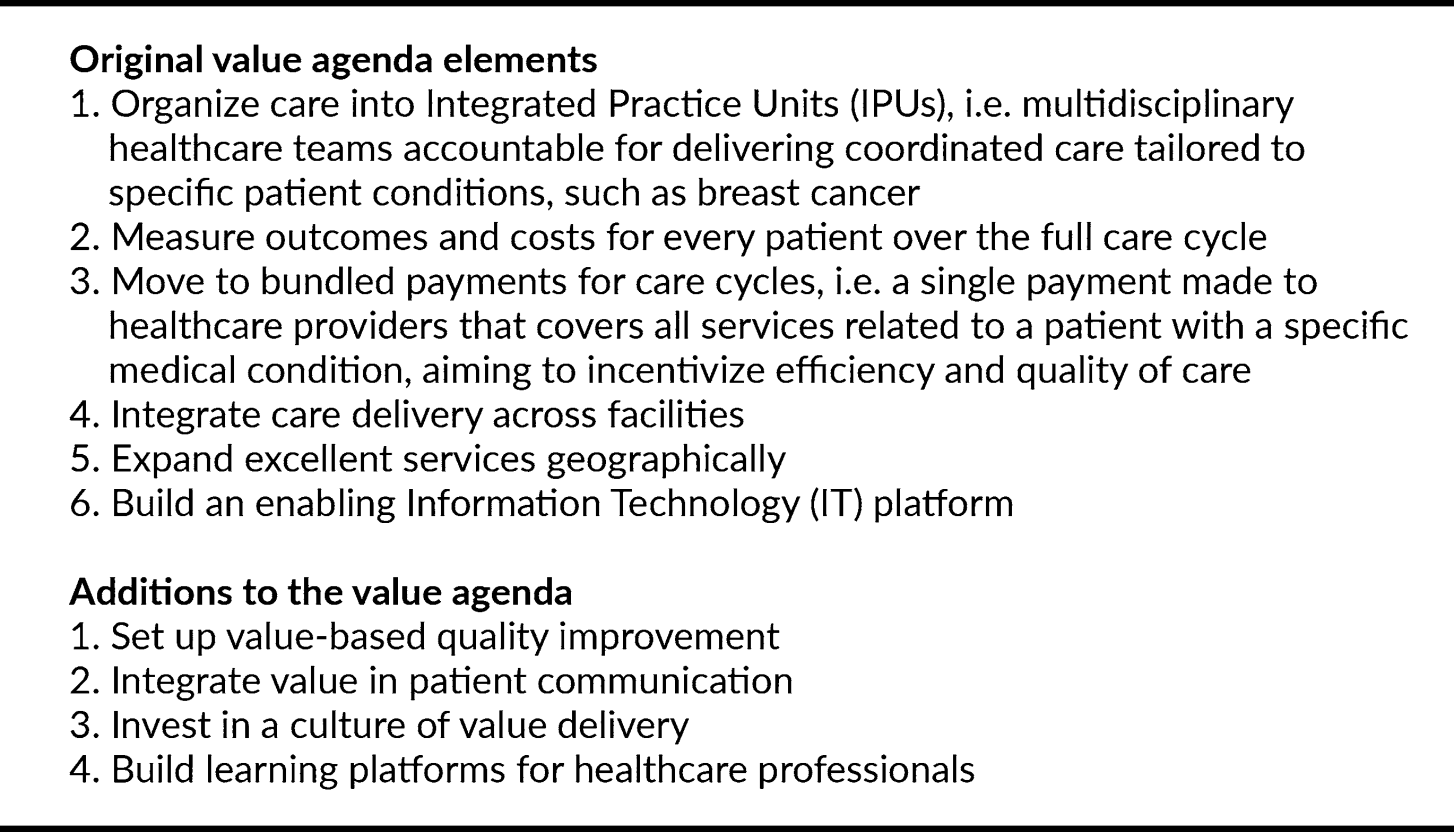
The six original value-agenda elements [5] and the four extensions [6]

Hospital have begun to move towards VBHC [9, 10], aligning with Porter and Lee’s emphasis on providers’ critical role in broader system reform: “*All stakeholders in health care have essential roles to play. […]. Yet providers must take center stage*” [11] (p. 70). VBHC adapts how contemporary healthcare is organized, delivered, and reimbursed, likely requiring (re)institutionalization and (re)professionalization [12, 13]. This is a complex endeavor due to its multifaceted, multi-level scope and the traditional resistance to change among medical professionals [13]. However, the value agenda lacks guidance on how hospitals can approach this, and literature lacks insights into hospitals’ strategies for implementing VBHC [3].

Research indicates that VBHC has been implemented partially thus far, initially focusing on either outcomes or costs but rarely both [3, 4, 10, 14–16]. Despite studies exploring implementation experiences and determinants [14, 17–25] and others suggesting roadmaps [26–29], detailed accounts of hospitals’ change processes are scarce [30]. Most studies have focused on initial experiences with local pilots, lacking long-term and organizational-level perspectives on change. Noteworthy exceptions include studies by Engels et al. [31] and Feitz et al. [32], which share experiences from a decade of value-based quality improvement implementation, and Bonde et al., studying the shift towards value-based governance [33].

Ramos et al. emphasized the importance of integrating complexity when implementing VBHC [14]. This approach builds on the increasing attention to embracing complexity in implementation [34–37], organizational change [38], and health services research [39], especially in inherently complex healthcare settings. Complexity thinking contrasts with linear, straightforward cause-and-effect approaches often associated with Implementation Science [35] and certain Change Management models [40]. Instead, it views change as fluid, resulting from multiple dynamics that cannot be fully overseen or managed. Complexity-informed research aims to unravel these dynamics and provide insights into what is happening and why [34, 40].

Despite the growing adoption of VBHC by hospitals, there remains a notable gap in understanding its implementation, particularly regarding rich, complexity-informed, organizational-level process studies. This retrospective, complexity-informed process study examines the decade-long (2012–2023) transition towards VBHC in a Dutch university hospital, Erasmus Medical Centre (Erasmus MC), aiming to partially close that gap. Specifically, this study aims to unravel how the hospital’s strategy to implement VBHC evolved and how implementation outcomes unfolded. Moreover, it aims to examine the logic behind these developments and provide stakeholder reflections on the process.

### National and hospital setting

**VBHC in The Netherlands**

In the Netherlands, VBHC currently focuses on the collection, use, and transparent reporting of outcomes data relevant to patients. This focus has been supported and guided by the Dutch government for the past 20 years [41], with impetus from a program on outcome-based care that ran from 2018 to 2022 [42]. In 2022, the “integral care agreement” [43] embraced VBHC as one of the four pillars. Moreover, it outlined two key ambitions to be realized by 2025: first, making outcome information publicly available for 50% of the disease burden, and second, routine use of these data by healthcare professionals to facilitate Shared Decision-Making (SDM) in consultations and improve quality. These ambitions respectively align with the value agenda extensions “integrate value in patient communication” and “set-up value-based quality improvement” [6]. SDM is perceived a component of VBHC in The Netherlands [44] and has been obligatory under Dutch law since 2021 [45].

Dutch hospitals are typically organized in specialty departments with informal multidisciplinary teams and operate in a market with regulated competition based on volume. There are experiments with adapting hospital structures (value agenda; element 1) [15], cost measurement (element 2) [46], and alternative payment methods (element 3) [31, 47]**.** The Netherlands lacks a centralized Electronic Health Records (EHR) system (challenge to element 6; enabling IT). Since 2017, a national learning network has connected patients, healthcare professionals, policymakers, and payers to facilitate knowledge and experience exchange regarding VBHC [48] (value agenda; addition 4).

**VBHC in Erasmus MC**

Erasmus MC is one of the largest Dutch university hospitals, with site details provided in Additional file 1. In 2012, alongside grassroots VBHC-related initiatives within the hospital, the Executive Board initiated exploration of VBHC’s potential [49]. Their interest was sparked when the Chief Executive Officer, invited by the founder of the VBHC Center Europe, attended a masterclass by Michael Porter at Harvard Business School. Earlier, internal consultants had gauged interest in the concept through open sessions, but this had not yet translated into concrete actions.

A Central Support Team (CST) coordinates and facilitates VBHC implementation. The CST grew from 1 full-time equivalent (FTE) in 2013 to approximately 6 FTEs in 2020, and has since been expanded with an integrated IT team. Two former physicians successively headed this team. In 2018, the Executive Board formed a steering committee. Patient are involved in implementation efforts as part of local improvement teams and a central panel. Since 2020, a separate team has been dedicated to international VBHC initiatives.

Throughout the hospital’s move to VBHC, there has been a focus on PROMs for clinical and shared decision-making, necessitating significant IT investments. This aligns with the government’s emphasis on patient outcomes and the hospital’s commitment to viewing the patient as a partner and leveraging the potential of data [50]. Specialty outpatients are asked to complete electronic PROMs before their outpatient consultation. The employed PROMs instruments are listed in Additional file 1.

## Methods

This complexity-informed [34–36, 51] process study [52] aims to retrospectively unravel how Erasmus MC’s strategy to implement VBHC evolved, how implementation outcomes unfolded, the logic behind these developments, and to provide stakeholder reflections on this matter. Examination spans from the start of implementation in 2012 to its status in 2023. Results are presented in a chronologically sequenced narrative [36, 52].

### Data sources

This study uses four data sources, including both existing data and newly collected data. Existing data included *documents,* and *implementation outcome indicators*. Data collection included a *survey* among clinicians, and *interviews* with individuals involved in the change to VBHC at the hospital level.

**Documents**

The first author received access to the CST’s online workspaces with 10,536 files spanning from 2012 to mid-2023. Files included implementation plans, evaluations, letters, minutes, and educational and communication materials, amongst others. The initial analysis comprised two-stages: (1) screening of all materials, resulting in the identification of 1564 documents containing data on strategies, logic, contextual factors, implementation outcomes, and reflections; and (2) examining these files and extracting data.

**Implementation outcome indicators**

We used four implementation outcome indicators [53, 54] from the hospital’s implementation monitoring system, which we labeled as follows: (1) *breadth*, i.e. the number of patients and (sub)departments participating in VBHC; (2) *depth*, i.e. the value agenda elements implemented; (3) *PROMs us*e, i.e. patients’ response rate to PROMs and clinicians’ use rates of the PROMs dashboard to view a patient’s response; and (4) *sustainment*, i.e. patients’ and (sub)departments’ continued participation in VBHC. The tracking of patients’ PROMs completion and clinicians’ use of the PROMs dashboard were automated, providing both daily and longitudinal scores, and could be filtered by department, type of PROM survey, and timespan. However, this extends beyond the scope of this study, which focuses solely on reporting aggregate rates. The other indicators were manually collected in a database by the CST.

**Survey**

A survey was digitally distributed to all 194 clinicians across the 35 (sub)departments that initiated PROMs implementation as a first step toward VBHC in January 2023, excluding one clinician who had been involved in survey design. Fifteen closed questions were posed (see Additional file 2), which were part of a larger survey (reference: EMC23). Two reminders were sent. After verifying the 57 responses, 47 were included in the analysis. Table 1 shows the reasons for exclusion and sample sizes for the different data sources.

**Interviews**

Twenty individuals contributing to VBHC at the hospital level were interviewed (see Table 1). The semi-structured interview questions centered on strategy as outlined in plans, its practical execution, explanations for potential discrepancies, and overall reflections. Participants were purposefully selected to include actors across the entire time span, relying on documents and snowballing. Two individuals refused participation for personal circumstances. The interviews were recorded, and transcribed verbatim.

**Table 1:** Data sources and sample sizes

Data source	Description		*N*
Documents (*n* = 10,536)	Files	Included	1564
Survey (*n* = 57)	Responses	Included	47
		Complete responses	42
		Excluded Demographic questions answered only (*n* = 5); Not providing patient care (*n* = 2); PROMs not yet available (*n* = 2); No familiarity with PROMs (*n* = 1)	10
	Sex	Female	35
	Age	Average in years (min, max)	46 (31, 64)
	Function	Medical specialist	30
		Nurse	12
		Other (e.g., psychologist, resident-in-training)	5
Interviews (*n* = 20)	Participants	Member Executive Board	1
		Director quality and patient safety	1
		Head VBHC (pre-)steering committee	2
		Member steering committee	2
		Lead CST	3
		Member CST	9
		External consultant	1
		Clinician in VBHC program	1
	Sex	Female	14
	Duration	Average in minutes	53

### Data analysis

**Fig. 1:**
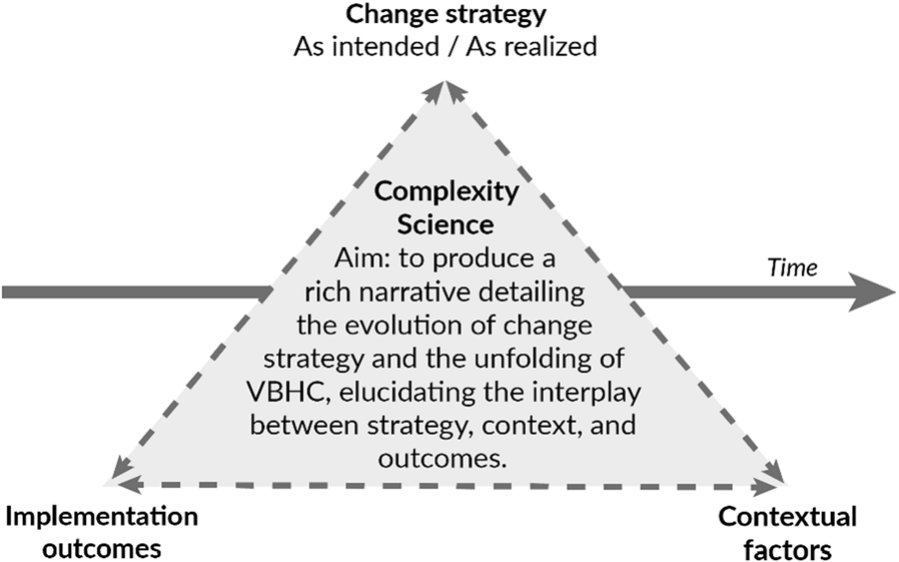
Data analysis

Guided by Langley’s work on analyzing process data [52], we used a three-step, iterative approach to construct a chronological narrative unraveling the evolution of VBHC and associated implementation strategy in Erasmus MC over past decade. Through the lens of complexity science [34–36, 51], we aimed to provide a nuanced account on how strategy, outcomes and contextual factors interact (see Fig. 1); thereby limiting oversimplification of reality.

- Step 1. Building the core strategy narrative
  From *document* data, primarily annual implementation plans, and enriched by *interview* data we extracted the VBHC elements intended for implementation, the targeted population, the envisioned timeline of change, and noted the year of the plan. This information was used to develop a chronologically sequenced narrative of how the intended strategy to implement VBHC evolved [52]. In parallel, from *document* data, primarily evaluations, and *interview* data we mapped how strategy was realized, i.e. the practical execution. “Strategy as intended” and “strategy as realized” are used as headers in the "Results" section.
  We identified strategy attributes using the factors of depth and breadth [55–58]. The depth factor assesses the extent of radical adaptation, focusing on the comprehensiveness of change in reference to the value-agenda and their integration in practice. The breadth factor evaluates the organizational scope of change, specifically measuring the degree of engagement of all patients and professionals, as well as the adaptation of organization-wide processes, policies, and systems. Both factors provide insight into the degree of (re-)institutionalization and (re-)professionalization around VBHC. Additionally, we draw inspiration from Maes and Hootegem’s typology for understanding various dimensions of change, including stride (incremental–revolutionary) and pace (slow–quick) [59].
- Step 2. Defining phases and adding implementation outcomes per phase
  We temporally bracketed [52] the narrative into phases based on significant shifts in intended strategy. The four *implementation outcome indicators* provided a snapshot of the implementation status at the end of each phase. These indicators required no further analyses. Additionally, we included the outcome sustainability [54], which captured stakeholders’ beliefs in the long-term endurance of VBHC, derived from *document* and *interview* data. This outcome is different from sustainment, which assesses whether implemented initiatives were continued.
- Step 3. Enriching the strategy narrative with logic and reflections
  Finally, we added information on the logic behind observed developments in the narrative and stakeholder reflections, derived from *document* and *interview* data. Data were open-coded and then axially coded into categories based on their shared topics using ATLAS.ti [60, 61]. *Survey* results were used to capture clinicians’ experiences with the transition to VBHC and their perceptions of the current VBHC implementation strategy. We examined and reported item-level frequencies.

## Results

Erasmus MC’s strategy to implement VBHC underwent two significant shifts over the decade. Initially, from 2014 to 2019, following a year of preparations, the aim was achieving deep, i.e. transformational, change by implementing multiple VBHC elements. Change efforts concentrated on small number of teams, supported by the CST (see “National and hospital setting”) After a one-year pilot among six teams, the CST and the Executive Board decided to continue this “*depth-first*” strategy, gradually expanding to other teams.

By 2020, implementation shifted into a multi-year, hospital-wide program, adopting a “*breadth-first*” strategy. This strategy aimed for large-scale, evolutionary change and initially focused on uniform implementation of PROMs across the entire hospital with integrated IT. Eventually, this strategy evolved into a *"hybrid”* strategy that delicately integrates both local, tailored and larger-scale, uniform changes, continuing into 2024.

**Fig. 2:**
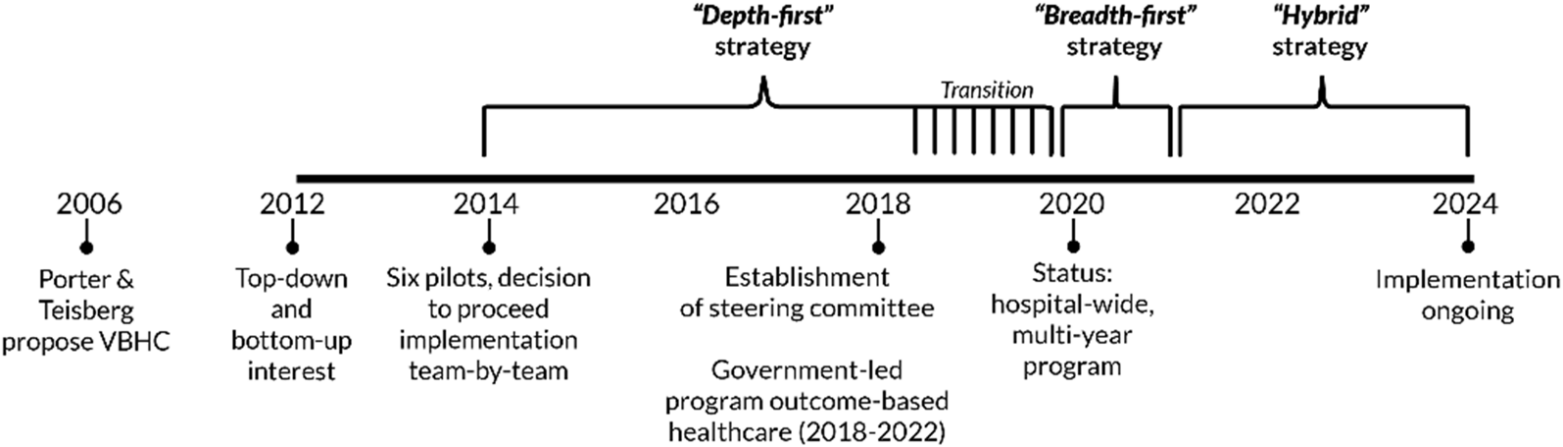
Timeline depicting the evolution of the strategy and key moments

Throughout these strategies, there has been a consistent focus on PROMs and their use in outpatient specialty consultations (value agenda; element 2 and addition 2). The change process evolved  organically, with the VBHC implementation strategy adapting based on accumulated insights and contextual developments, seizing opportunities as they arose. Figure 2 outlines the change process, including some key contextual factors described in “National and hospital setting”.

On average, clinicians rated the implementation process of PROMs 5.4 out of 10 and implementation outcomes 4.9 out of 10 (both min 1, max 9), with no significant differences among those commencing implementation across both strategies. Despite the hospital conducting research on VBHC initiatives [29, 62–74] (see Additional file 3), it had not yet quantitatively examined the impact of VBHC initiatives across the hospital on patient outcomes and costs. Additionally, the impact on the workforce remained unknown. This has become a growing concern, both to maintain investment and convince skeptics. Interviewee 1 expressed: *“Despite our strong belief in it, there comes a point where we need to provide evidence of its impact, especially considering the substantial investment of resources.”* This is echoed by an internal document dated 20/5/19, stating “*There is a need to determine the tangible benefits of VBHC, not only for the patient but also financially.”*

In the remainder of the “Results” we discuss the “depth-first” strategy (“Depth-first” strategy) and the “breadth-first” strategy turning into “hybrid” strategy (“Breadth-first” strategy). For each, we discuss key contextual factors, implementation as intended, implementation as realized, outcomes, and reflections. In “Towards a shift in strategy”, we describe the phase that bridges the “depth-first” and “breadth-first” strategies.

### “Depth-first” strategy

**Context**

During the years 2014–2019, interviewees encountered several challenges that hindered the success of VBHC*.* While the Executive Board verbally supported VBHC, their commitment varied with changes in board composition. Interviewee 15 remarked, *“The Executive Board did not fully give the green light for the movement we were making.”* The need to request a budget annually created insecurity and required significant time and effort. According to interviewees, the building of a new hospital building (2009–2018) and change of Electronic Health Record (EHR) provider in 2017 diverted attention and resources. Interviewee 14 regretted that the Executive Board did not use the opportunity of the new building to structure the hospital around medical conditions instead of siloed disciplines (value agenda; element 1). Interviewee 9 partially attributed this caution to reorganization issues faced by a Swedish hospital implementing VBHC [75]. The CST also faced limitation from PROMs and supportive IT not yet being available. The team’s capacity (see “National and hospital setting”) and the lack of IT support consistently bottlenecked progress, resulting in waiting lists for (sub)departments seeking to initiate VBHC implementation and compromised implementation support (internal document 20/5/19).

**“Depth-first”: strategy as intended**

Together with an external consultant, the CST developed a plan outlining how informal, multidisciplinary teams overseeing all care around a patient condition, such as cleft lip, could implement VBHC with their assistance. The focus was on achieving deep change by implementing numerous elements of the value agenda (see Fig. 4, quadrant A). They would assist a few teams at a time, providing tailored support and applying learnings from earlier trajectories to new teams, gradually expanding until VBHC was implemented for all patient conditions.

Initial steps in the team-level plan aimed at fostering collective understanding of VBHC, selecting PROMs, and defining appropriate measurement moments in the care path. These sessions would involve representatives from the clinical team, patients, and the CST. Next, the clinical team would measure PROMs among outpatients a few days prior to their consultation using an online survey and discuss patients’ responses during their appointment (value agenda; element 2 and addition 2). Moreover, they would measure costs through Time-Driven Activity-Based Costing [76] (also element 2). Subsequently, after approximately nine months, the team would use the aggregated PROMs data to drive value-based quality improvements (value agenda; addition 1). To support these activities, three tools were to be developed: an electronic PROMs survey system, a consultation room dashboard displaying a patient’s PROM outcomes and another for improvement purposes displaying aggregated PROMs data (element 6). Other VBHC elements such as networked care (element 4), benchmarking (part of addition 1) and bundled payment (element 3) were not integrated in this plan but were anticipated to be addressed in subsequent steps or on request. Ultimately, the vision was: “*To give clinicians the feeling that they collectively operate their own shop. […]. A shop that can promote its services to insurers, patients, and other medical facilities, emphasizing its commitment to delivering exceptional value”* (interviewee 14). At the organizational level, middle management would undergo VBHC training (addition 3).

**“Depth-first”: strategy as realized**

The above-mentioned three tools were developed, and teams started using PROMs in their outpatient specialty practice. Certain teams were supported to implement additional elements of the value agenda, e.g. bundled payment, however, without concurrently adhering to the initial plan.

Unforeseen circumstances prompted two additions to the abovementioned team-level plan. First, due to limited availability of PROMs, multiple teams were compelled to contribute to the development of PROMs, e.g., [64, 77–85], causing delays but fostering support for the content of PROMs. These efforts extended to the development of Patient Reported Experience Measures [86, 87]. Second, it became evident that care pathways were often either missing or outdated, requiring significant revamping efforts. This presented an opportunity for making initial care pathway improvements, yielding benefits in the eyes of clinicians. Additionally, three training sessions were developed, one of which trained clinicians in discussing PROMs with outpatients.

Four key aspects of the initial plan were not realized as intended. First, cost measurement was discontinued due to challenges in accurately assessing costs, e.g., allocating square meter prices and costs of assistive personnel to patients with specific conditions. Financial intricacies in the university hospital, involving funds for education and research, heightened the complexity. Moreover it was indicated that “*prioritizing quality as the starting point for change facilitated clinician engagement”* (interviewee 9). Second, PROM-informed care improvement activities occurred less frequently than anticipated due to limited IT support, constraints on workforce time, and suboptimal data quality. Third, among the first teams, the intended nine-month timeframe was not met due to the initial development of tools taking several years, causing disappointment and frustration. Fourth, training for department heads and managers was discontinued at their request, resulting in limitations in their support to clinical teams. Reasons included perceived theoretical abstraction and a mismatch with the trainer’s style. In 2018, the implementation plan was adjusted to accelerate the implementation of PROMs using generic items, initiating the shift towards the “breadth-first” strategy.

**Implementation outcomes in 2019**

In 2019, the outcomes achieved could be characterized as semi-deep and relatively narrow in breadth (see Fig. 4, quadrant B). Thirty-eight teams out of more than 200 were in the process of implementing electronic PROMs, of which ten achieved PROMs measurement and sustained this practice up to 2020, with eight continuing into 2024. Ten teams paused implementation due to capacity issues or challenges in team functioning. Additionally, one department implemented PROMs independently of the CST’s central VBHC efforts. Some teams implemented additional VBHC elements next to PROMs (see Table 2). Yet, by the end of the study, no team implemented all elements in the value agenda.

**Table 2:** Implementation outcomes in 2019

Outcome	Topic		*N*
Depth and breadth	Enabling IT	# teams with infrastructure	10 and 1 department
	PROMs	# teams collecting PROMs	10 and 1 department
		# teams preparing implementation	28
		# unique outpatients to whom PROMs were sent	36,135
	Care pathway improvement	# teams, not-based on PROMs data	38
		# teams, PROMs data-informed	10
	Cost measurement	# teams	3
	Benchmarking	# teams	3
	Networked care	# teams	2
	Bundled payment	# teams	1

In total, PROMs were distributed to 36,135 unique outpatients, with the majority (22,737 unique outpatients) involving the department that implemented PROMs independently. The monitoring system’s data indicated limitations in patients’ *use of PROMs,* i.e. their compliance in responding. Anecdotal data showed variations in clinicians’ *use of PROMs* during outpatient specialty consultations, with some always using them and others never. A more detailed exploration of these topics falls outside the scope of this study.

The *sustainability* of implementation, i.e., predicting long-term endurance, faced limitations, as described in the section below. In 2019, apart from cost measurement, VBHC initiatives were s*ustained*, indicating the actual continuity of implementation. Most of these initiatives continued through 2024, except for the PROM-informed care improvement activities, which were halted shortly after the strategy shift in 2020 and are expected to be restarted in 2024.

**“Depth-first” strategy: reflections**

Some interviewees appreciated the emphasis on deep implementation by incorporating multiple elements of the value agenda, accommodating diverse clinician interests and ambitions, and providing various learning opportunities. Furthermore, this approach aimed not only to adapt how care is delivered, but also how it is organized and reimbursed. This comprehensive approach was considered essential for achieving and sustaining change by aligning all forces. However, there were concerns about overwhelming conservative professionals, as many clinicians already find using PROMs challenging, as noted especially by interviewee 10.

The approach of implementing VBHC among informal multidisciplinary teams was deemed crucial for VBHC by some (see also the limitations of a departmental approach described in the next section). However, it also posed challenges related to reliance on team functioning and the varying support and motivation from both colleagues and department heads in specialty departments. Interviewee 3 exemplified this: “*In surgery, there were one or two of those VBHC teams. But they had many colleagues who were not involved, lacked understanding, and lacked belief in VBHC. These colleagues depicted these teams as if they were a group of hobbyists.*” Further, the Executive Board expressed dissatisfaction with the limited reach despite substantial investments. Some clinical teams served relatively small patient populations, prompting questions about whether to prioritize conditions with larger patient volumes or continue with the most enthusiastic clinical teams. However, the lack of data on patient volumes by care path hindered prioritization based on such information.

Moreover, the tailored, localized approach resulted in “*[…] a surge of local, enthusiasm-driven initiatives”* (interviewee 12). While enhancing the fit of solutions and local actors’ ownership, this approach faced drawbacks. Interviewees mentioned fragmented implementation efforts, conflicting local visions, lack of critical mass and absence of a stable overarching strategy. The developed IT saw advances yet had limitations, not optimally laying the groundwork for other value agenda elements. Each team had its own customized PROM-solution developed, leading to a proliferation of PROMs and IT applications, for which there was neither enough funding nor workforce for development and maintenance. Further, this situation hindered cross-departmental data analysis and collaboration, and imposed a burden on multimorbid patients to complete multiple overlapping surveys. Additionally, clinicians encountered limitations from PROMs not being EHR-integrated.

Taken together, the tailored, team-focused approach hindered scaling and posed risks to sustainability. Notably, an internal document (8/12/2013), showed that many of these limitations were foreseen at the start. The proposed solutions, such as integrating PROMs in the EHR and the use of generic PROMs, appear to have gained feasibility and acceptance only at a later stage.

### Towards a shift in strategy

The year 2019 was primarily dedicated to evaluating and reorienting change, led by an internal consultant. The shortcomings of the “depth-first” change phase led to disappointment, waning patience, and a loss of credibility in the initial VBHC implementation strategy across various organization layers. An internal document (20/5/2019) states: *“collaboration on multiple fronts—strategic, tactical, and operational—has not been successful everywhere, resulting in current noise regarding the topic and the future vision of VBHC.”* Another document, dated 22/5/2019, states: “*It is not a pilot project but rather a cultural shift, yet it remained stuck in the pilot phase*.” Nevertheless, prior achievements motivated a commitment to advancing VBHC, anticipating benefits from expanding its reach, and taking it to a higher level of maturity: “*After the initial pioneering phase, there is a need for structure. There is a need to* i*mplement and sustain VBHC from a strategic, hospital-wide standpoint.”* Interviewee 3 explained that successful change necessitates a delicate balance between local, and centralized efforts: *“It is nice to see that enthusiasm, but there must also be a counterweight to it. If VBHC is completely determined by people who are extremely passionate about working with outcomes, then one dies in beauty. […]. However, it should not just become very practical and managerial either, turning it into a cold, soulless program.”*

In the lead-up to professionalizing VBHC, in 2018, the Executive Board formed a steering committee to address buy-in challenges among major stakeholders such as IT, department heads and clinicians. The formation of the steering committee was “*a kind of rescue*” (interviewee 4) as it “*[…] assigned a leadership role to several people, increasing their engagement*” (interviewee 5). Yet, one member of the steering committee reflected: *“I am not sure if we actually steer. It is primarily an information exchange platform”* (interviewee 2). Although the CST suggested the Executive Board to head this steering committee (internal document 20/5/2019), a department head who had independently achieved PROMs implementation in their department was appointed as the head.

This person’s belief in evolutionary change, starting with PROMs, along with the desire to approach change from a hospital-wide perspective, and contextual factors such as the development of generic PROMs, contributed to shifting the strategy from “depth-first” to “breadth-first.” Despite some disagreement from the former VBHC head, the Executive Board approved the new strategy, designating it as a multi-year, hospital-wide program starting in 2020.

### “Breadth-first” strategy

**Context**

National attention for VBHC strengthened (see “National and hospital setting”), and there was improved availability of PROM instruments. As VBHC became a hospital program, the CST extended to include an integrated IT team. However, the capacity of the CST continued to pose a consistent bottleneck in progress. While financial resources transitioned from annual budget allocations to multi-year funding, internal documentation (22/12/2022) indicates that financial constraints still led to scaled-down plans. Similarly to before, no dedicated resources to implement VBHC were made available to (sub)departments, although they also did not face direct monetary costs associated with VBHC implementation.

The Executive Board expressed verbal support for VBHC, although perceptions of its adequacy varied among interviewees. Starting in 2022, their involvement extended to requiring (sub)departments to formally report on their VBHC activities and acknowledging those that performed well. COVID-19 prompted exploration of new applications of VBHC principles, for example as a triage tool for the limited operating room capacity [88–90]. Nonetheless, this initially encountered resistance from some, as it could potentially lead to loss of revenues, and later lost urgency as the COVID-19 situation stabilized.

**“Breadth-first”: strategy as intended**

The “breadth-first” strategy aimed to incrementally implement VBHC across the entire hospital (see Fig. 4, quadrant C). Contrary to the previous focus on informal, multidisciplinary teams around patient conditions, implementation advanced through the traditional structure of (sub)departments, tackled a few at a time, if they showed interest. There was a central belief in simplifying implementation for clinicians, unifying tooling and embedding change in the hospital’s systems and policies. As a result, the role and power of the CST expanded, diminishing front-line clinicians’ involvement, and significant effort went into professionalizing IT.

**Fig. 3:**
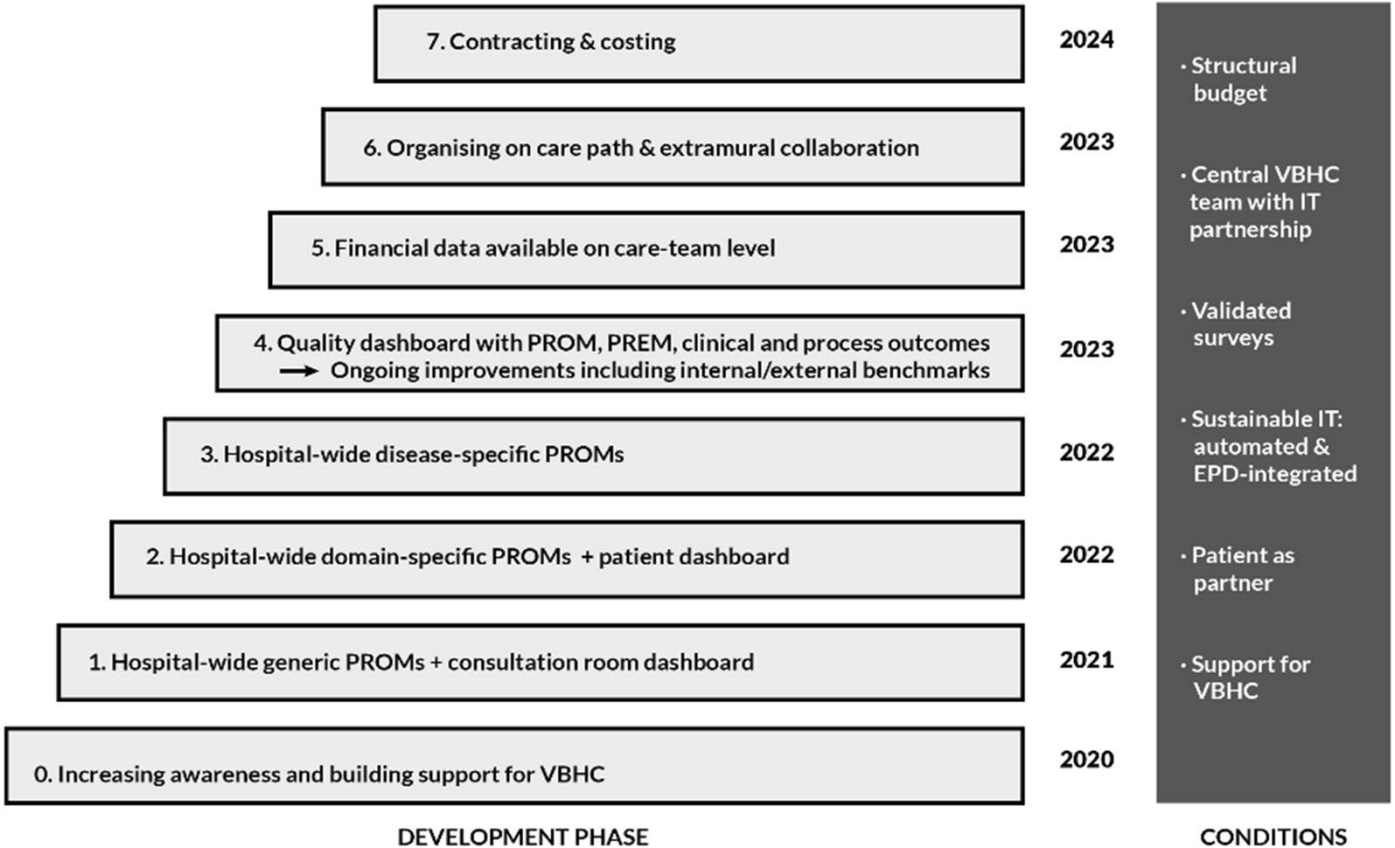
The “breadth-first” strategy plan (translated from internal document Annual Plan 2023)

Implementation was guided by an organization-level, multi-year plan (2020–2024) that consisted of eight sequential steps to be executed over a five-year period (see Fig. 3). Although this plan appears quite straightforward, interviews uncovered nuances, less linearity, and uncertainties. The first three years would focus on VBHC knowledge promotion and the implementation of three-tiered, EHR-integrated PROMs (value agenda; elements 2 and 6). In 2020, the first step was to homogenously implement generic PROMs (tier 1) throughout the entire hospital, encompassing questions related to daily functioning and quality of life. The underlying idea was that this standardized set could rapidly enable the entire hospital to measure PROMs, immediately presenting opportunities to enhance the quality of patient consultations (value agenda; addition 2). These generic PROMs (tier 1) would be complemented by domain-specific PROMs in 2021 (tier 2), measuring outcomes relevant for specific patient groups, and eventually tailor-made, disease-specific PROMs in 2022 (tier 3). According to interviewees prioritizing generic PROMs was resource-driven, rather than the ideal for patients and clinicians. Additional value agenda elements were scheduled for 2023 and 2024, yet detailed plans for these were not disclosed. The creation of Integrated Practice Units (value agenda; element 1) was considered inappropriate in several cases because of small patient populations for rare diseases and the hospital’s complex organizational and financial structures.

**“Breadth-first”: strategy as realized**

Although the content of the plan remained largely the same and was acted upon, there were seven notable changes in the timing, and order. Overall, these changes indicate a departure from the linear progress presented in Fig. 3.

**Fig. 4:**
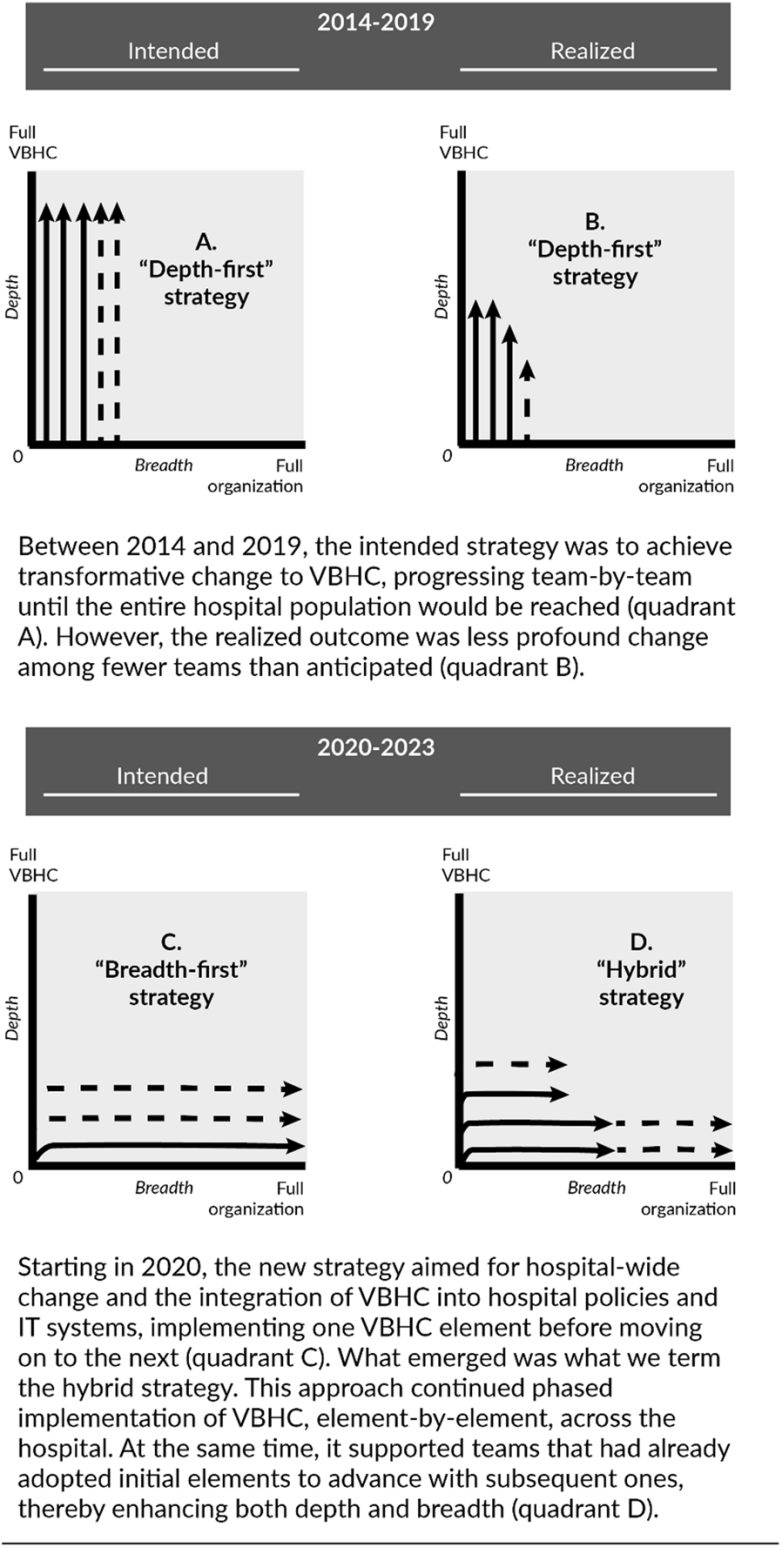
The evolvement of strategy, as intended and as realized, along the dimensions of depth and breadth. The arrows symbolize implementation efforts

First, (sub)departments increasingly requested the complete three-tiered PROMs set, rather than waiting for the hospital-wide implementation of generic PROMs before moving on to domain and disease-specific PROMs. Survey results, interviews, and documents emphasize that generic PROMs often did not provide enough benefits to clinicians and patients. Interviewee 15 noted: “*generic does not do justice to the complexity inherent in an academic setting.*” Implementing complete PROM sets required customization, which subsequently slowed down the expansion to larger populations. Second, contrary to the initial plan for homogeneous change, there was increased heterogeneity in implementation. The strategic plan dated 20/03/2023, refers to the adoption of a “hybrid” strategy. In this “hybrid” strategy, the CST combined uniform, larger-scale approaches with tailored, local approaches. The goal was to advance hospital-wide implementation of generic PROMs and integrate VBHC in the hospital system, while simultaneously provide support to several (sub)departments to adopt disease-specific PROMs and deepen their VBHC implementation through subsequent value-based interventions (see Fig. 4, quadrant D). For example, teams will start PROMs-informed quality improvement in 2024. Moreover, a new cost measurement pilot is attempted, guided by the belief “*in the healthcare crisis that is unfolding, we cannot avoid addressing the costs*” (interviewee 3). Third, the planned development of a dashboard for patients to review their PROM outcomes was postponed due to the hospital-wide development of a smartphone application, where this feature is intended to be integrated. According to interviewee 6, the current absence hindered patients’ active engagement. Fourth, change fell behind on the extended schedule. Fifth, despite the delay, various unplanned activities were undertaken. These “spin-offs” were in response to workforce requests or external developments, like COVID19. For example, clinicians requested the use of the PROMs’ IT infrastructure to inquire about patients’ medication and lifestyle. Additionally, PROMs were included as a metric for triage. Sixth, documents indicated that unforeseen IT challenges caused considerable PROMs dashboard loading times due to data accumulation, prompting several clinicians to stop discussing PROMs. This required additional attention to resolve and promote clinicians’ re-uptake, thereby compromising implementation outcomes. Last, in response to limitations in clinicians’ use of PROMs, the CST began offering on-the-job coaching on how to discuss PROMs. This effort was deemed necessary in addition to other training resources like a manual and courses.

**Implementation outcomes in 2023**

In 2023, the VBHC adoption status is diverse, with some departments starting to embrace VBHC more deeply, while others have yet to initiate it. In December 2023, 68 (sub)departments collected PROMs among their outpatients, of which 50 implemented a complete, three-tiered PROM, i.e., generic, domain-specific and disease-specific. In November 2023, 12,335 PROMs, with separate tallies per PROM, were sent to 5107 unique outpatients. This is 17% of all outpatients, and is a conservative estimate for two reasons: it incorporates duplicate PROMs registrations from canceled appointments, and not all outpatients are eligible for PROMs participation. Ineligible are patients seeking acute or psychiatric care or a second opinion, those with a one-stop-shop appointment, certain patients with intellectual disability, neurodiversity, and specific selections determined by (sub)departments. Patients’ and clinicians’ *use of PROMs* fell below expectations (see Table 3), despite initiating several interventions to enhance this. Investigation into this matter falls outside the scope of this study. Overall, the VBHC implementation was deemed increasingly *sustainable* (see section below). Regarding actual *sustainment*, results indicate that two (sub)departments quit using PROMs due to a shift in the patient treatment policy, moving towards a one-time visit without follow-up consultations.

**Table 3:** Implementation outcomes at the end of 2023

Outcome	Topic		*N*
Depth and breadth	Enabling IT	# teams with infrastructure	Hospital-wide
	PROMs	# (sub)departments collecting PROMs	68
		% outpatients reached (see note in main text)	17
		# PROMs sent in total	278,269
		# PROMs sent monthly	> 10,000
PROMs use	Patients	# PROMs completed 07/2020-12/2023	≈ 123,000
		% response rate 07/2020-12/2023 (average)	43
		% response rate 12/2023 (average)	52
	Clinicians	% patient responses opened in dashboard 07/2022-12/2023 (average)	17
		% patient responses opened in dashboard 12/2023 (average)	15
Sustainment	Patients	% patients discontinuing PROMs use	Data missing
	Clinicians	# (sub)departments discontinuing PROMs	2

**“Breadth-first” strategy: reflections**

The focus on hospital-wide change enabled the adaptation of core policies and systems and facilitated communication through hospital-wide channels. Further, associated uniformity and standardization streamlined IT implementation. Yet, overreliance on uniformity and standardization introduced limitations, such as diminished local-fit and a sense of ownership among local sites. Further, decision-making authority and responsibilities increasingly shifted to the CST, placing an additional burden on their limited capacity. Interviewee 3 reflected on the diversity among clinicians, noting the need for complementary use of local, tailored implementation efforts that allow for heterogeneity: *“Some people are very enthusiastic about VBHC, hoping for a swift and comprehensive implementation, while others have reservations and are pleased with the slower, phased process we follow.”* Several interviewees believed a core strength lied in the eventually adopted “hybrid” strategy that enabled both tailoring to match local sites’ interests and needs along with coherence and system integration, improving sustainability.

Perceptions regarding the prioritization of PROMs were mixed. Interviewee 11 clarified the rationale for commencing with PROMs before other value agenda elements: *“One creates a slippery situation when changing the care pathway first or when altering it during the collection of baseline data. Ensuring the availability of patient outcome data is crucial to assess the impact of modifications made to the care pathway.”* However, interviewee 20 expressed doubt: *“eliminating inefficiencies from your process may not always result in an immediate improvement in patient outcomes […] However, it could potentially lead to benefits like cost reduction or increased efficiency”*, emphasizing that it is crucial to include various outcomes.

In contrast, interviewee 17 disliked this priority, perceiving that the concentrated focus on outpatient use of PROMs limited behavior change: “*It is not just the dialogue with the patient that nurtures the culture, absolutely. But the collaborative effort to enhance care serves as the other culture nurturer*.” Interviewee 16 reflects “*we did not consider the effects of focusing on one VBHC element while pausing or neglecting the others. […] In my opinion, this was no longer in balance.*” Moreover, the narrow focus overlooked the perverse incentives associated with the prevailing healthcare system, such as volume-based payment. Interviewee 18 encountered conflicting messages, needing to prioritize value but occasionally being asked to increase volume once again. Documents described similar issues, such as the inability to simplify a care pathway due to payments being linked to specific steps. Moreover, while implementation among (sub)departments enhanced scalability, increased collegial understanding, and improved patient volumes, potentially facilitating clinicians to adopt new routines, it simultaneously raised concerns. Interviewee 17 and 14 respectively described: “*[…] clinicians still manage their personal responsibilities within the confines of their own consultation rooms while VBHC is about taking collective responsibility for the entire care path.”* and “*Focusing solely on one’s own discipline limits the potential impact on enhancing patient outcomes, rendering PROMs less relevant*”. Overall, these issues raised concerns that the initial “breadth-first” strategy could potentially lead to VBHC becoming *“a wrongly loaded concept or an empty shell*” (interviewee 17).

Nonetheless, in general, interviewees appreciated the newly developed (IT) foundation, with some anticipating it * to function as a catalyst”* (interviewee 19). The combined VBHC-IT team was considered a strength. Survey responses indicated that 45% of the clinicians (*n* = 19) endorsed hospital-wide change, 40% (*n* = 17) supported phased implementation, and 38% (*n* = 16) prioritized outpatient PROMs use, highlighting mixed perceptions.

Regarding healthcare professionals’ motivation, limitations emerged due to the extended time for the implementation: “*One can’t keep clinicians engaged and maintain momentum for five years*” (interviewee 6). Furthermore, interviewees noted constraints stemming from a lack of perceived urgency for change and the absence of disincentives for non-adherence: “*There is no fire. There are no patients dying if you don’t use PROMs*” (interviewee 11). Some clinicians perceived themselves as already working in a value-based manner prior to VBHC (survey respondent 39) or believed it would be a passing trend (survey respondent 11). Interviewees also noted limitations regarding the lack of evidence and the terminology around VBHC, with “value” sometimes being associated with a monetary focus (interviewee 11). Inconsistent framing and policy competition were highlighted as sources of confusion and change fatigue (interviewee 3).

With the implementation experience obtained thus far, some change actors desired an immediate hospital-wide rollout of generic PROMs with increased Executive Board mandate. Others endorsed the current phased strategy of cultivating enthusiastic adopters and tailored implementation support. Interviewee 19 stated: “*It has to come from the right motivation, not just because there is a checkbox to be ticked.*”.

## Discussion

This retrospective, complexity-informed process study unraveled the decade-long (2012–2023) transition towards VBHC at Erasmus MC. It explored how the hospital’s strategy to embrace VBHC evolved, how implementation outcomes unfolded, and the underlying logic behind these developments. We found that achieving the healthcare transformation intended by VBHC requires moving beyond siloed and linear theories on change. Instead, integrated and complexity-informed approaches [34, 35] seem necessary to successfully (re)institutionalize and (re)professionalize [12] according to the VBHC paradigm [1, 6, 11] as ultimate aims.

### The evolvement of implementation strategy

Erasmus MC adopted a data-driven, patient outcome focused approach to VBHC, emphasizing the electronic capture of PROMs among outpatients and the discussion of individual patients’ responses during their outpatient specialty consultations. PROMs appear to act as “functional pressure” [91], enabling clinicians to adapt their roles to VBHC by integrating holistic information about patients’ experienced symptoms, functioning, and quality of life. This operationalization of VBHC aligns with the extended “value agenda” [6]. While we cannot claim a direct cause-effect relationship, this focus is consistent with the Dutch government’s emphasis on patient outcomes [41–43], the obligation of SDM under Dutch law [45], and the hospital’s mission to position the patient as a partner [50].

Over the course of a decade, Erasmus MC’s strategy to implement VBHC evolved from what we termed “depth-first” to “breadth-first,” and eventually to a “hybrid” strategy. Depth refers to the level of transformative change, while breadth refers to the scope of organization-wide change [55–58]*.* Initially, the focus was on deep change through local, tailored implementation of multiple VBHC elements. The strategy then transitioned to a hospital-wide program aimed at evolutionary change on a large scale, emphasizing the integration of VBHC into mainstream IT and policies. For example, PROMs were integrated into the EHR and VBHC was gradually formalized through its integration into mandatory reporting cycles for departments. This reduction in depth has also been observed in other VBHC-implementations [3, 4, 21, 31, 92]. While both strategies yielded successes, they also had limitations. Therefore, the hybrid strategy aimed to delicately combine deep and broad change efforts.

The strategy evolved organically throughout the decade, diverging from linear-looking plans. Change was facilitated and coordinated by the CST, as recommended [26], which was later effectively extended with an integrated IT team. They adapted the VBHC implementation strategy based on accumulated insights and contextual developments, seizing opportunities as they arose. The CST navigated challenges including financial limitations and uncertainties, as well as their VBHC initiatives outpacing external advancements like PROMs development and payment reform. Additionally, the strategy evolved as implementation matured and decision-makers changed, underscoring complexity arising from individuals holding differing values regarding the move to VBHC [93].

**Combining depth and breadth focused strategy**

**Fig. 5:**
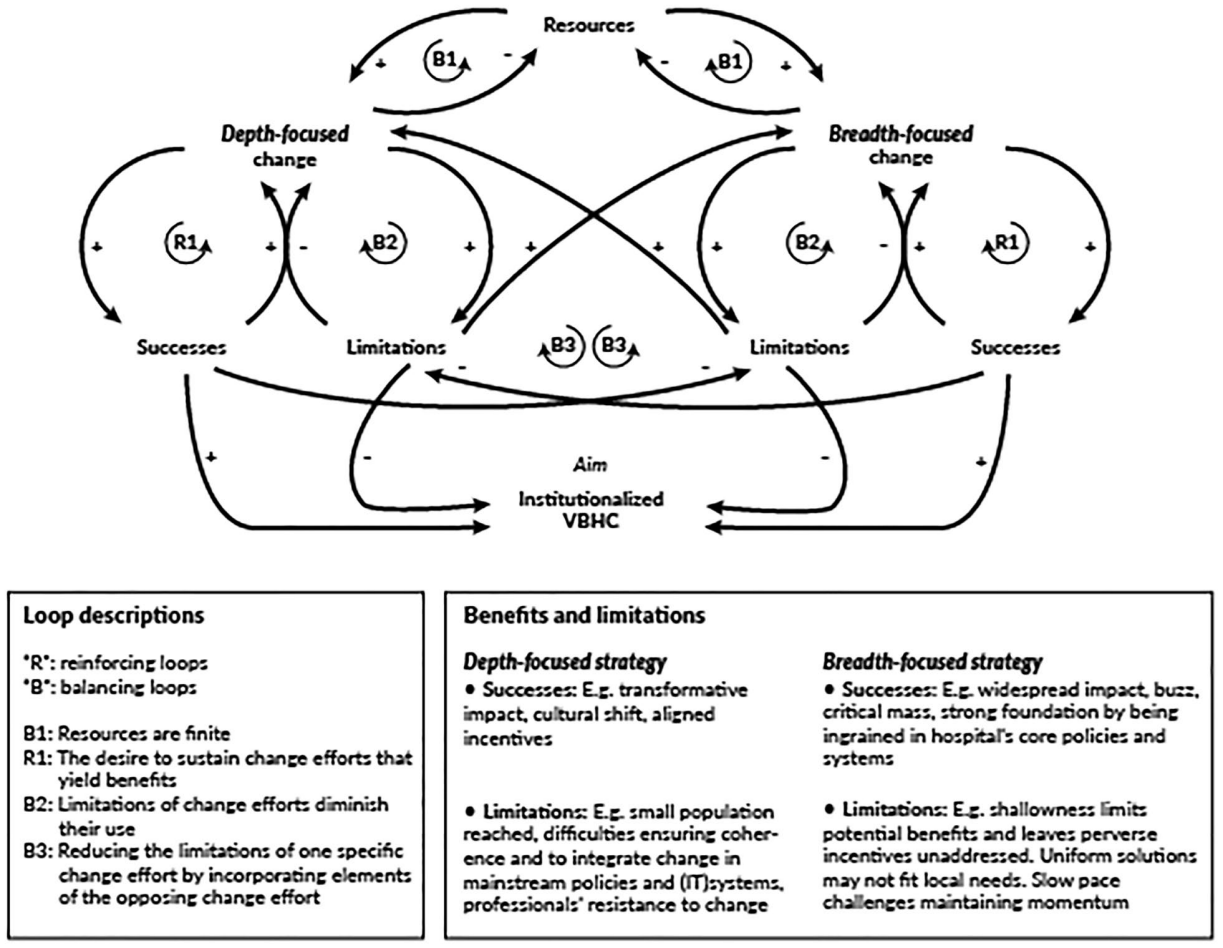
A causal loop diagram illustrating key dynamics in “depth-focused” and “breadth-focused” change

Combining a strategy that balances “depth-focused” and “breadth-focused” change seems crucial for achieving and institutionalizing VBHC. Figure 5 illustrates this delicate equilibrium using a causal loop diagram [94], showing reinforcing and balancing forces, labeled “R” and “B.”

The loops titled “B1” highlight the competing demands for resources. Our findings reveal a strategic choice between allocating resources to facilitate transformative change for a few individuals or fostering incremental progress across the entire hospital. The remainder of the causal loop diagram demonstrates that both depth-focused and breadth-focused strategies contribute positively to VBHC institutionalization. However, each approach also brings its own limitations, which necessitate resolution through the opposing strategy.

For example, deep change efforts may face challenges such as lack of coherence, inadequate integration in organization-wide processes, insufficient support by peers, and slow scalability. These issues can be addressed through broader actions. Conversely, broad change initiatives may be criticized for their superficiality, uniformity, and slower development in depth, potentially resulting in VBHC becoming an ‘empty shell.’ To counter these limitations, it is crucial to complement broad initiatives with localized, in-depth efforts (loops B3).

These findings underscore the tension between deep and broad change, demonstrating that deep change cannot be uniformly imposed on a large scale [58, 95, 96]. Instead, large-scale deep change appears to emerge as the cumulative outcome of at the organizational level facilitated and coordinated local, deep change trajectories. Therefore, VBHC is not easily scalable, and its implementation poses a challenge of balancing both types of efforts.

**Implementation outcomes**

This study provides additional evidence of partial VBHC implementation, focusing more on patient outcomes than costs [3, 4, 10, 14–16]. Moreover, it reveals significant diversity in the hospital’s adoption status, with some departments embracing VBHC more deeply than others. Over the decade, progress was hampered by capacity constraints of the CST, resulting in waiting lists to start VBHC.

In 2023, PROMs implementation reached 68 (sub)departments and, as a conservative estimate, 17% of all outpatients. Each month, more than 10,000 electronic PROMs are sent, and clinicians are supported by a consultation room dashboard for discussing PROM outcomes with patients. Clinicians expressed moderate satisfaction with both the process and outcomes of implementing PROMs. The developed (IT) foundation is poised to spearhead subsequent efforts in value-based quality improvement. Additionally, stemming from the depth-first strategy, a few teams pioneered networked care, benchmarking initiatives, and bundled payments. However, the hospital’s efforts have mostly lacked an extramural focus, and care has remained organized around disciplines and reimbursed based on volume so far.

Achieving satisfactory patient response to PROMs and clinicians’ acknowledgment of this data proved challenging. This is concerning because their behaviors ultimately determine the success of VBHC, even if PROMs are recognized as tools. Limitations may be due  to suboptimal facilitation and difficulties in (re)professionalization. Our findings suggest that clinicians may not perceive a strong enough sense of urgency for change to prompt immediate action or disrupt habitual ways of working, while such urgency is considered critical in various change theories [97, 98]. They may also be hindered by current conditions and institutional complexity (see below). Another factor could be healthcare professionals’ existing belief that they already deliver value-based care and not necessarily see the benefit of using PROMs for this purpose. Nonetheless, the heightened focus on VBHC may have initiated gradual behavioral shifts among healthcare professionals, such as a greater emphasis on patient priorities and resource allocation. However, further studies are needed to validate this assumption of micro-level institutionalization processes [98].

**Institutional complexity**

From our findings, we note challenges of institutional complexity, where individuals confront  institutional logics that prescribe different norms and behaviors [99]. This complexity appears to hinder the institutionalization of VBHC. For instance, professionals are expected to work value-based while still being paid based on volume. Additionally, VBHC inherently seems to hold levels of institutional complexity, asking professionals to simultaneously consider patient outcomes and costs. When resources are limited, this could create value conflicts, such as deciding whether to prioritize those in highest need, equity, or achieving the greatest value for society [100].

VBHC not only imposes changes on professionals’ work but also relies on them to drive the transformation [101]. However, healthcare professionals’ contemporary competences and attitudes, i.e. their professionalization, may not align with the demands of driving and thriving in VBHC [13, 102]. In our study, we observed a limitation: characteristics of complex change, such as unpredictability, uncertainty about outcomes, and the need for experiential learning, were not fully integrated into plans, stakeholder communications and training. This oversight may have contributed to unwarranted expectations and limited stakeholder engagement [95].

**Recommendations for practice**

One should not waste time trying to define the ultimate strategy to implement VBHC, as this is illusory. As others have noted, there seems no “good” or “bad” strategy for VBHC [103, 104]. It seems important to avoid overly linear approaches and limit dichotomous thinking. Instead, adapt based on continuous learning and co-evolving conditions [59, 105, 106]. We recommend integrating knowledge from diverse theoretical schools on implementation and change, striving for the higher-level aims of (re)institutionalization and (re)professionalization [12]. Achieving and institutionalizing VBHC requires investments in both systems and people, supported by transformational leadership [107] and sponsorship at all levels. One may benefit from integrating VBHC into all operations rather than treating it as a separate initiative, and capitalizing on the expertise, energy, and creativity of the workforce. These investments should be sustained, recognizing that cultural shifts and new practices typically require significant time to take root [108]. Change agents could familiarize themselves with the different pathways to institutional change [98], mechanisms to propel change [57], and the concept of complexity [34, 35, 93].

A critical question revolves around VBHC’s impact on the medical and nursing profession: how does VBHC and its implementation align with or challenge contemporary values, role identities, and capabilities of healthcare professionals? The answer seems contingent on how VBHC is operationalized. What is expected of healthcare professionals in VBHC? Are they tasked to achieve what matters to individual patients, engage in SDM, provide inclusive care, oversee and collaborate in patients’ full care cycle, enhance prevention, evaluate interventions not only in relation to outcomes but also in terms of their costs, and so on? How are these role identities and capabilities structurally integrated into medical and nursing education and demonstrated by role models in practice? Similarly, we have limited knowledge on how to cultivate and sustain a workforce capable of driving and thriving in care transformations and evolving professions, such as VBHC.

A deeper understanding about what VBHC, and associated concepts like high-value, cost-conscious care (HVCCC), imply for practice [74, 109], along with studies on their alignment with and implications for education [110–112] are needed. Helpful resources include a tool to evaluate HVCCC attitudes [113] and support for developing change capability [114], medical leadership [115] and nurse leadership [116]. Above all, aligned with complexity thinking [34–36, 51], every actor has a role to play in (the journey to) VBHC, and no one can truly oversee and manage the entire process.

### Strengths and limitations

Limitations include that this study is focused on a Dutch university hospital, which context may differ from other healthcare organizations. The local conceptualization of VBHC, which is impartial and emphasizes two extensions to the original value agenda, may differ from how other organizations operationalize and approach VBHC. Nevertheless, we believe that several insights provided by this study transcend specific value-agenda elements and may hold true for complex change in general. Our focus on organizational-level change represents just one element in the broader chain of actors. Avenues for future research include embracing individual and team levels, leadership, the broader healthcare context, education, and the interplay among these factors. Methodologically, linear models do not fit well when studying complex change, and it should be acknowledged that conclusions on effective strategies are often impossible since outcomes are frequently not attributable to a single cause and outcomes like culture change take time to manifest [108].

Regarding data, document and implementation outcomes data rely on analyses conducted by the CST, potentially introducing bias. Interviewees’ accounts may be influenced by recall bias. The low survey response rate (29%) is a limitation, although the high variation in respondents’ satisfaction suggests the inclusion of clinicians with both positive and negative opinions. The timing of the survey followed a period of IT challenges in using PROMs, potentially affecting results. Finally, the implementation outcome indicators on patient responses to PROMs and professionals’ use of the PROMs dashboard serve as proxies rather than capturing the actual value derived from improvements in the quality of conversations. We regret that limitations in the hospital’s data analytics have constrained us from integrating data such as patients’ experiences with the care they received. Addressing these constraints in data access and connectivity is crucial, as it is essential for facilitating research on the impact of VBHC.

Notwithstanding these limitations, this study contributes to bridging the gap in the literature on how to achieve VBHC in hospital-setting [3]. Through our long-term, organization-level, complexity-informed study design, our work offers a distinctive contribution to the existing literature, surpassing the scope of local pilot studies and studies oversimplifying change by lacking attention to non-linear dynamics.

## Conclusion

Insights from a decade of VBHC implementation in a Dutch university hospital suggest that VBHC does not lend itself to linear planning and is not easily scalable. There appears to be no golden standard for change. Rather, achieving the transformation intended by VBHC requires moving beyond siloed theoretical schools on change. It necessitates an adaptive and delicate approach that combines “depth” and “breadth” focused efforts, underpinned by transformational leadership and sponsorship at all levels. Local, deep changes facilitated and guided at both organizational and system levels culminate in large-scale transformation. Embracing complexity and focusing on the ultimate aims of (re)institutionalization and (re)professionalization are crucial. At the core of this endeavor lies the imperative to sustain this transformative journey collectively, driven by capability, opportunity, and motivation.

### Supplementary Information

Additional file 1: Information about Erasmus MC.

Additional file 2: Survey.

Additional file 3: VBHC literature with Erasmus MC authorship.

## Data Availability

The interview and survey data are available from the corresponding author upon reasonable request.

## Abbreviations

VBHC: Value-based health care
PROMs: Patient reported outcome measures
Erasmus MC: Erasmus Medical Centre
SDM: Shared decision making
CST: Central Support Team
FTE: Full-time equivalent
IT: Information Technology
EHR: Electronic Health Record
PREMs: Patient Reported Experience Measures
IPU: Integrated Practice Unit
