# Supplementary material for: A decade of change towards Value-Based Health Care at a Dutch University Hospital: a complexity-informed process study
Source: Health Res Policy Syst. 2024 Aug 5;22:94. doi: 10.1186/s12961-024-01181-z (PMC11301982; doi:10.1186/s12961-024-01181-z)

**Additional file 1. Information about Erasmus Medical Center and their VBHC program**

Information about Erasmus Medical Center (data from 2022)

| Hospital type | University hospital, including a pediatric hospital  Private not-for-profit |
| --- | --- |
| Work organization | Specialty departments are dominant with informal interunit multidisciplinary teams |
| Mission | A healthy population and excellent care through research and education |
| Vision | Recognized as a leader in innovations for health and care |
| Ambitions (2019-2023) | 1. Distinctive innovation, focus on technology and data  2. Attention for employee and organization  3. Positioning the patient as a partner |
| Total # employees | 14,700 |
| - Of which female | 73% |
| - Of which physicians | 6% |
| Beds | 1,200 |
| Admissions yearly | 30,300 |
| Outpatient consultations yearly | 629,000 |
| Unique patients yearly | 187,000 |
| Revenue yearly | 2,2 billion |
| Billing | All healthcare professionals are hospital employees, i.e. pay-roll employees |

**Reference:** PricewaterhouseCoopers Accountants N.V. (2022). *Jaarverslaglegging 2022 [annual report 2022]*.

**PROMs used by Erasmus MC**

| **Tier** | **Type** |
| --- | --- |
| Generic | PROMIS v1.2 Global Health |
| Domain Specific | PROMIS SF v2.0 Physical function 4a  PROMIS SF v1.0 Anxiety 4a  PROMIS SF v1.0 Depression 4a  PROMIS SF v1.0 Fatigue 4a  PROMIS SF v1.1 Pain interference 4a  PROMIS SF v1.0 Sleep disturbance 4a  PROMIS SF v1.0 Satisfaction with participation in social roles 4a |
| Disease-specific | Various |

**VBHC at Erasmus MC poster summary (internal document dated 17/08/2023)**


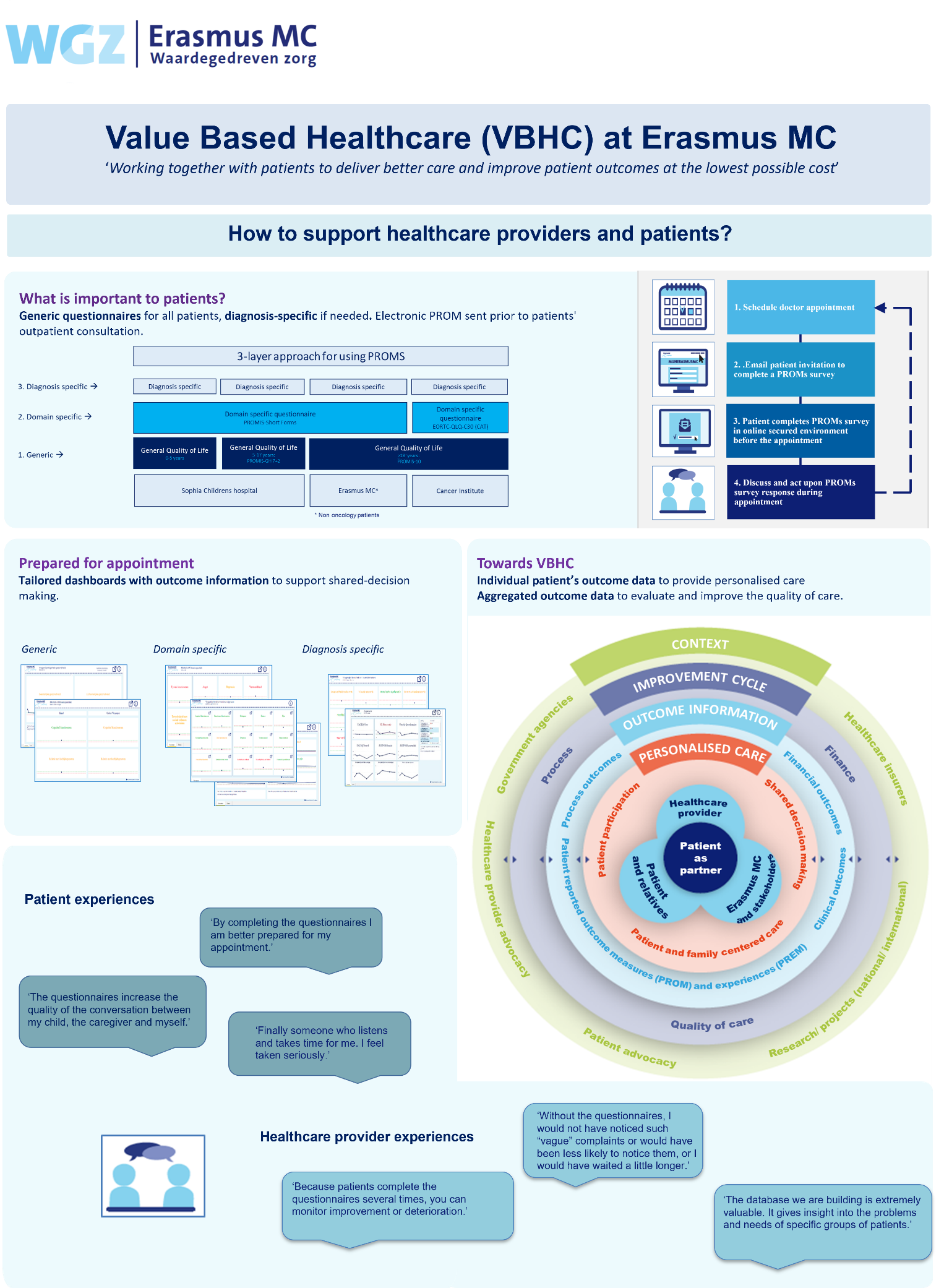

Supplement: Supplementary file 1 — Additional file 1: Information about Erasmus MC. [file 12961_2024_1181_MOESM1_ESM.docx]
