# Supplementary material for: A decade of change towards Value-Based Health Care at a Dutch University Hospital: a complexity-informed process study
Source: Health Res Policy Syst. 2024 Aug 5;22:94. doi: 10.1186/s12961-024-01181-z (PMC11301982; doi:10.1186/s12961-024-01181-z)
Supplement: Supplementary file 2 — Additional file 2: Survey. [file 12961_2024_1181_MOESM2_ESM.docx]

**Additional file 2. Survey**

The survey encompassed questions designed for multiple studies. The items below are specific to this manuscript. Legend: majority, i.e. 50% or more of respondents selected this answer, is color coded green.

| Item | Answer categories | n responses | % | |
| --- | --- | --- | --- | --- |
| 1. Gender | Male | 47 | 26 | |
|  | Female |  | 47 | |
|  | Other |  | 0 | |
| 1. Age | Average | 47 | 46 years | |
|  | Max |  | 64 years | |
|  | Min |  | 31 years | |
|  | StdDev |  | 8,3 years | |
| 1. Function | Medical Specialist | 47 | 64 | |
|  | Doctor in training |  | 4 | |
|  | Nurse specialist |  | 11 | |
|  | Nurse |  | 9 | |
|  | Nurse consultant |  | 6 | |
|  | Psychologist |  | 4 | |
|  | Sexologist |  | 2 | |
| 1. Year work experience (excluding main education) | 2 - 5 years | 47 | 4 | |
|  | 6 - 10 years |  | 9 | |
|  | 11 - 25 years |  | 62 | |
|  | More than 25 years |  | 26 | |
| 1. To what implementation program (phase) do you belong? | I am part of a pioneer team for VBHC: My team initiated the use of PROs (Gemstracker or Zorgmonitor) from 2013 to 2019 | 47 | 38 | |
|  | I am part of the hospital-wide VBHC program: PROs were implemented in my department between 2020 and 2022 |  | 34 | |
|  | I don't know |  | 28 | |
| 1. Which PROMs have been implemented at your workplace? | I don't know | 45 | 18 | |
|  | Generic PROMs and/or domain-specific PROMs |  | 29 | |
|  | Disease-specific PROMs |  | 16 | |
|  | Both generic/domain specific and disease specific PROMs |  | 38 | |
| 1. Were you present during PROMs implementation at your workplace? | Yes | 47 | 77 | |
|  | No, I started working there later |  | 13 | |
|  | I don’t know |  | 11 | |
| 1. How frequently do you examine patients’ responses to PROMs? | I have never used PROs | 47 | 11 | |
|  | I have stopped looking at PROs |  | 15 | |
|  | Occasionally |  | 36 | |
|  | As often as possible |  | 21 | |
|  | Always |  | 17 | |
| 1. *If in the previous question either answer 3, 4 or 5 was selected:*   To what extent do you discuss these outcomes with the patient during the consultation? | Never | 35 | 17 | |
|  | Occasionally |  | 29 | |
|  | As often as possible |  | 26 | |
|  | Always |  | 29 | |
| 1. Al in all, what grade (1-10) do you give to the process of PROMs implementation in your consultation room? | Average | 44 | 5,4 | |
|  | Min |  | 1 | |
|  | Max |  | 9 | |
|  | StdDvt |  | 2,4 | |
| 1. Al in all, what grade (1-10) do you give the outcomes of implementing PROMs in your consultation room? | Average | 44 | 4,9 | |
|  | Min |  | 1 | |
|  | Max |  | 9 | |
|  | StdDvt |  | 2,3 | |
| 1. Which of the following VBHC activities have also been part of your work? (Multiple answers possible) | Forming and working in a multidisciplinary team for a specific condition | 43 | 56 | |
|  | Redesigning care pathways (within the hospital) |  | 30 | |
|  | Learning, improving, and/or innovating based on PROs data and/or benchmarks |  | 33 | |
|  | Actions related to integrated care |  | 21 | |
|  | Actions related to healthcare costs and financing |  | 9 | |
|  | None of the above activities, and I also do not aspire to do so |  | 9 | |
|  | None of the above activities, although I do have an interest in them |  | 23 | |
| 1. I support Erasmus MC's choice to implement VBHC in phases, starting with PROMs (instead of one of the activities mentioned above). | Disagree | 42 | 17 | 24 |
|  | Partly disagree |  | 7 |  |
|  | Neutral |  | 36 | |
|  | Partly agree |  | 21 | 40 |
|  | Agree |  | 19 |  |
|  | No opinion |  | 0 | |
| 1. I believe it is important for Erasmus MC to focus on hospital-wide implementation of PROMs | Disagree | 42 | 21 | 29 |
|  | Partly disagree |  | 7 |  |
|  | Neutral |  | 24 | |
|  | Partly agree |  | 17 | 45 |
|  | Agree |  | 29 |  |
|  | No opinion |  | 2 | |
| 1. I believe it is important for Erasmus MC to explicitly encourage the use of PROMs in the outpatient care consultation room. | Disagree | 42 | 19 | 26 |
|  | Partly disagree |  | 7 |  |
|  | Neutral |  | 36 | |
|  | Partly agree |  | 10 | 38 |
|  | Agree |  | 29 |  |
|  | No opinion |  | 0 | |
