# Supplementary material for: A decade of change towards Value-Based Health Care at a Dutch University Hospital: a complexity-informed process study
Source: Health Res Policy Syst. 2024 Aug 5;22:94. doi: 10.1186/s12961-024-01181-z (PMC11301982; doi:10.1186/s12961-024-01181-z)
Supplement: Supplementary file 3 — Additional file 3: VBHC literature with Erasmus MC authorship. [file 12961_2024_1181_MOESM3_ESM.docx]

**Additional file 3. VBHC literature with Erasmus MC authorship**

On February 28th, 2023, we systematically analyzed seven databases to retrieve international peer-reviewed articles from the Dutch university hospital Erasmus Medical Center (Erasmus MC) regarding Value-Based Health Care (VBHC). The search was conducted with support of W. Bramer, a librarian from Erasmus Medical Center, on February 28th, 2023.

**Search string**

The search string contained words related to “*VBHC*” , as well as the hospital’s developed VBHC dashboard which they initially called “*Healthcare Monitor*” next to either “*Erasmus*” or the location being “*Rotterdam*”.

**Embase 133**

('value based care'/de OR 'value based medicine'/de OR (vbhc OR vb-hc OR value-based OR valuebased OR ((high-value OR value-driven) NEAR/3 (care OR healthcare)) OR healthcare-monitor OR health-care-monitor):ab,ti) AND (erasmus* OR rotterdam):ab,ti,ad

**Medline 4**

(Value-Based Health Care/ OR (vbhc OR vb-hc OR value-based OR valuebased OR ((high-value OR value-driven) ADJ3 (care OR healthcare)) OR healthcare-monitor OR health-care-monitor).ab,ti,kw.) AND (erasmus* OR rotterdam).ab,ti,kw,ia.

**psycINFO 2**

((vbhc OR vb-hc OR value-based OR valuebased OR ((high-value OR value-driven) ADJ3 (care OR healthcare)) OR healthcare-monitor OR health-care-monitor).ab,ti.) AND (erasmus* OR rotterdam).ab,ti.

**Web of science 154**

TS=(((vbhc OR vb-hc OR value-based OR valuebased OR ((high-value OR value-driven) NEAR/2 (care OR healthcare)) OR healthcare-monitor OR health-care-monitor))) AND ALL=(erasmus* OR rotterdam)

**CINAHL 3**

((MH Value-Based Health Care+ OR TI(vbhc OR vb-hc OR value-based OR valuebased OR ((high-value OR value-driven) N2 (care OR healthcare)) OR healthcare-monitor OR health-care-monitor)) OR AB((vbhc OR vb-hc OR value-based OR valuebased OR ((high-value OR value-driven) N2 (care OR healthcare)) OR healthcare-monitor OR health-care-monitor))) AND (erasmus* OR rotterdam)

**Business Source Premier 18**

((MH VALUE-based healthcare OR MH VALUE-based management OR TI(vbhc OR vb-hc OR value-based OR valuebased OR ((high-value OR value-driven) N2 (care OR healthcare)) OR healthcare-monitor OR health-care-monitor)) OR AB((vbhc OR vb-hc OR value-based OR valuebased OR ((high-value OR value-driven) N2 (care OR healthcare)) OR healthcare-monitor OR health-care-monitor))) AND (erasmus* OR rotterdam)

**EconLit 7**

TI,AB((vbhc OR vb-hc OR value-based OR valuebased OR ((high-value OR value-driven) N/2 (care OR healthcare)) OR healthcare-monitor OR health-care-monitor)) AND (erasmus* OR rotterdam)

**Search outcomes and analysis**

The search yielded 198 items (see Table 1), of which 15 articles were included after abstract and full-text screening, using four exclusion criteria: 1) not about VBHC; 2) Erasmus MC not the empirical field; 3) not a full article; and 4) participation in a Delphi study for PROMs outcome set development, e.g. [1–10]. The selected articles were then categorized and described based on their scope. We identified 15 peer-reviewed VBHC articles from the hospital, inductively categorized into four topics: VBHC implementation, VBHC operationalization, PROMs implementation, and the utilization of PROMs as a data source, see Table 2.

**Table 1**. Search outcomes per database

| **Database searched** | **Platform** | **Years of coverage** | **Records** | **Records after duplicates removed** |
| --- | --- | --- | --- | --- |
| Embase | Embase.com | 1971 - Present | 133 | 130 |
| Medline ALL | Ovid | 1946 - Present | 4 | 0 |
| Web of Science Core Collection* | Web of Knowledge | 1975 - Present | 154 | 57 |
| CINAHL* | EBSCO | 1982 - Present | 3 | 0 |
| PsycINFO | Ovid | 1806 - Present | 2 | 0 |
| Business Source Premier | EBSCO | 1922 - present | 18 | 8 |
| EconLit | ProQuest | 1886 - present | 7 | 3 |
| **Total** | | | **321** | **198** |

*Science Citation Index Expanded (1975-present) ; Social Sciences Citation Index (1975-present) ; Arts & Humanities Citation Index (1975-present) ; Conference Proceedings Citation Index- Science (1990-present) ; Conference Proceedings Citation Index- Social Science & Humanities (1990-present) ; Emerging Sources Citation Index (2005-present). No other database limits were used than those specified in the search strategies

**Table 2**. Identified articles categorized in four themes

| Theme | Details | References |
| --- | --- | --- |
| VBHC implementation | Collaboration with multiple institutions | [11] |
| VBHC operationalization | Consensus on value-based outpatient consultations among clinicians | [12] |
| PROMs implementation | Central evaluation among all participating clinicians in VBHC | [13] |
|  | Decentral evaluation among specific disciplines, including patient experiences with PROMs and perceived service outcomes | [14, 15] |
|  | Collaboration with multiple institutions | [11, 16, 17] |
| PROMs as data source | Specific to diseases or disciplines | [3, 18–24] |

**References**

1. Ong WL, Schouwenburg MG, Van Bommel ACM, et al (2017) A standard set of value-based patient-centered outcomes for breast cancer: The International Consortium for Health Outcomes Measurement (ICHOM) initiative. JAMA Oncol 3:677–685. https://doi.org/10.1001/jamaoncol.2016.4851

2. Nijagal MA, Wissig S, Stowell C, et al (2018) Standardized outcome measures for pregnancy and childbirth, an ICHOM proposal. BMC Health Serv Res 18:953. https://doi.org/10.1186/s12913-018-3732-3

3. Mulder J, Galema-Boers AMH, de Jong-Verweij LM, et al (2020) The development and first results of a health-related outcomes set in familial hypercholesterolemia (FH) patients: Knowledge is health. Atherosclerosis 293:11–17

4. Allori AC, Kelley T, Meara JG, et al (2017) A standard set of outcome measures for the comprehensive appraisal of cleft care. Cleft Palate-Craniofacial Journal 54:540–554. https://doi.org/10.1597/15-292

5. van Balen EC, O’Mahony B, Cnossen MH, et al (2021) Patient-relevant health outcomes for hemophilia care: Development of an international standard outcomes set. Res Pract Thromb Haemost 5:1–13. https://doi.org/10.1002/rth2.12488

6. Kampstra NA, Grutters JC, Van Beek FT, et al (2019) First patient-centred set of outcomes for pulmonary sarcoidosis: A multicentre initiative. BMJ Open Respir Res 6:. https://doi.org/10.1136/bmjresp-2018-000394

7. Kim AH, Roberts C, Feagan BG, et al (2018) Developing a Standard Set of Patient-Centred Outcomes for inflammatory Bowel Disease-an international, cross-disciplinary consensus. J Crohn’s Colitis 12:408–418. https://doi.org/10.1093/ecco-jcc/jjx161

8. Hummel K, Whittaker S, Sillett N, et al (2021) Development of an international standard set of clinical and patient-reported outcomes for children and adults with congenital heart disease: A report from the International Consortium for Health Outcomes Measurement Congenital Heart Disease Working Group. Eur Heart J Qual Care Clin Outcomes 7:354–365. https://doi.org/10.1093/ehjqcco/qcab009

9. Akpan A, Roberts C, Bandeen-Roche K, et al (2018) Standard set of health outcome measures for older persons. BMC Geriatr 18:36. https://doi.org/10.1186/s12877-017-0701-3

10. Fierens L, Carney N, Novacek G, et al (2023) A Core Outcome Set for Inflammatory Bowel Diseases: Development and Recommendations for Implementation in Clinical Practice Through an International Multi-stakeholder Consensus Process. J Crohns Colitis 1–13. https://doi.org/10.1093/ecco-jcc/jjad195

11. Cossio-Gil Y, Omara M, Watson C, et al (2022) The Roadmap for Implementing Value-Based Healthcare in European University Hospitals—Consensus Report and Recommendations. Value Health 25:1148–1156. https://doi.org/10.1016/j.jval.2021.11.1355

12. van Engen V, Bonfrer I, Ahaus K, Buljac-Samardzic M (2023) Identifying consensus on activities that underpin value-based healthcare in outpatient specialty consultations, among clinicians. Patient Educ Couns 109:107642. https://doi.org/10.1016/j.pec.2023.107642

13. Amini M, Oemrawsingh A, Verweij LM, et al (2021) Facilitators and barriers for implementing patient-reported outcome measures in clinical care: An academic center’s initial experience. Health Policy (New York) 125:1247–1255. https://doi.org/10.1016/j.healthpol.2021.07.001

14. Dronkers EAC, Baatenburg de Jong RJ, van der Poel EF, et al (2020) Keys to successful implementation of routine symptom monitoring in head and neck oncology with “Healthcare Monitor” and patients’ perspectives of quality of care. Head Neck 42:3590–3600. https://doi.org/10.1002/hed.26425

15. van Egdom LSE, Lagendijk M, van der Kemp MH, et al (2019) Implementation of Value Based Breast Cancer Care. European Journal of Surgical Oncology 45:1163–1170. https://doi.org/10.1016/j.ejso.2019.01.007

16. Laureij LT, Been J V., Lugtenberg M, et al (2020) Exploring the applicability of the pregnancy and childbirth outcome set: A mixed methods study. Patient Educ Couns 103:642–651. https://doi.org/10.1016/j.pec.2019.09.022

17. Apon I, Rogers-Vizena CR, Koudstaal MJ, et al (2022) Barriers and Facilitators to the International Implementation of Standardized Outcome Measures in Clinical Cleft Practice. Cleft Palate-Craniofac J 59:5–13

18. Sreeram II, ten Kate CA, van Rosmalen J, et al (2021) Patient-Reported Outcome Measures and Clinical Outcomes in Children with Foregut Anomalies. Children-Basel 8:

19. Huberts AS, Clarijs ME, Pastoor H, et al (2023) Sexual well-being in patients with early-stage breast cancer at 1- and 2-year follow-up. J Sex Med. https://doi.org/10.1093/jsxmed/qdad007

20. Oemrawsingh A, van Leeuwen N, Venema E, et al (2019) Value-based healthcare in ischemic stroke care: case-mix adjustment models for clinical and patient-reported outcomes. BMC Med Res Methodol 19:229. https://doi.org/10.1186/s12874-019-0864-z

21. Depla AL, Lamain-De Ruiter M, Laureij LT, et al (2022) Patient-Reported Outcome and Experience Measures in Perinatal Care to Guide Clinical Practice: Prospective Observational Study. J Med Internet Res 24:. https://doi.org/10.2196/37725

22. van den Hoven AT, Bons LR, Dykgraaf RHM, et al (2020) A value-based healthcare approach: Health-related quality of life and psychosocial functioning in women with Turner syndrome. Clin Endocrinol (Oxf) 92:434–442. https://doi.org/10.1111/cen.14166

23. Popping S, Kall M, Nichols BE, et al (2021) Quality of life among people living with HIV in England and the Netherlands: a population-based study. The Lancet Regional Health - Europe 8:100177. https://doi.org/10.1016/j.lanepe.2021.100177

24. Van Zijl FVWJ, Lohuis PJFM, Datema FR (2022) The Rhinoplasty Health Care Monitor: Using Validated Questionnaires and a Web-Based Outcome Dashboard to Evaluate Personal Surgical Performance. Facial Plast Surg Aesthet Med 24:207–212. https://doi.org/10.1089/fpsam.2020.0549
